# The C1q and gC1qR axis as a novel checkpoint inhibitor in cancer

**DOI:** 10.3389/fimmu.2024.1351656

**Published:** 2024-04-22

**Authors:** Berhane Ghebrehiwet, Michal Zaniewski, Audrey Fernandez, Mathew DiGiovanni, Tiana N. Reyes, Ping Ji, Anne G. Savitt, Jennie L. Williams, Markus A. Seeliger, Ellinor I. B. Peerschke

**Affiliations:** ^1^ Department of Medicine, Stony Brook University, Stony Brook, NY, United States; ^2^ Department of Pathology, Stony Brook University, Stony Brook, NY, United States; ^3^ Department of Microbiology and Immunology, Stony Brook University, Stony Brook, NY, United States; ^4^ Department of Pharmacology, Stony Brook University, Stony Brook, NY, United States; ^5^ Department of Laboratory Medicine, Memorial Sloane Kettering Cancer Center, New York, NY, United States

**Keywords:** gC1qR, receptor for the globular “heads” of C1q, GHA, globular head A chain, C1QBP, the gene for gC1qR

## Abstract

Understanding at the molecular level of the cell biology of tumors has led to significant treatment advances in the past. Despite such advances however, development of therapy resistance and tumor recurrence are still unresolved major challenges. This therefore underscores the need to identify novel tumor targets and develop corresponding therapies to supplement existing biologic and cytotoxic approaches so that a deeper and more sustained treatment responses could be achieved. The complement system is emerging as a potential novel target for cancer therapy. Data accumulated to date show that complement proteins, and in particular C1q and its receptors cC1qR/CR and gC1qR/p33/HABP1, are overexpressed in most cancer cells and together are involved not only in shaping the inflammatory tumor microenvironment, but also in the regulation of angiogenesis, metastasis, and cell proliferation. In addition to the soluble form of C1q that is found in plasma, the C1q molecule is also found anchored on the cell membrane of monocytes, macrophages, dendritic cells, and cancer cells, via a 22aa long leader peptide found only in the A-chain. This orientation leaves its 6 globular heads exposed outwardly and thus available for high affinity binding to a wide range of molecular ligands that enhance tumor cell survival, migration, and proliferation. Similarly, the gC1qR molecule is not only overexpressed in most cancer types but is also released into the microenvironment where it has been shown to be associated with cancer cell proliferation and metastasis by activation of the complement and kinin systems. Co-culture of either T cells or cancer cells with purified C1q or anti-gC1qR has been shown to induce an anti-proliferative response. It is therefore postulated that in the tumor microenvironment, the interaction between C1q expressing cancer cells and gC1qR bearing cytotoxic T cells results in T cell suppression in a manner akin to the PD-L1 and PD-1 interaction.

## Introduction and background history

According to the available global statistics, cancer is the leading cause of death worldwide, accounting for nearly 10 million deaths in 2022 globally, with breast and lung cancers leading the way. By 2030, it is projected that there will be 26 million new cancer cases worldwide and 17 million cancer deaths per year. However, despite treatment advances made over the last decade, cancer recurrence and metastasis still represent the most important global public health challenge. Therefore, identification of novel therapeutic modalities directed against diverse and novel tumor targets are needed if we are to develop tumor specific combination therapies that will deepen disease response and improve patient outcomes. Although there is ample evidence showing that the complement system plays an important role in carcinogenesis and metastasis, research directed at targeted understanding of the mechanism by which complement as a system, or its individual components, participate in the pathology of cancer is desperately lacking. Among the complement proteins identified to have strong potential as targets for cancer therapy are: C1q and the receptor for the globular heads of C1q designated gC1qR (1). Both C1q and gC1qR have been shown to be expressed in a wide range of healthy cells but overexpressed in many cancer types including triple negative breast cancer (1). Because the C1q molecule, which is overexpressed on cancer cells, and gC1qR, which is expressed on tumor infiltrating T cells, have the potential to function in a manner similar to the PD-L1 and PD-1 checkpoint inhibition, the C1q-gC1qR axis is emerging as an important novel target for the development of therapy against most if not all cancer cells. However, before we proceed to validate the potential of “gC1qR” as a target candidate for cancer therapy, it is important to first highlight the history and the sequence of events that led to the discovery of this fascinating molecule so as to clarify some of the confusions that are often associated with the use of three or sometimes four names for the same molecule.

The discovery of the “32/33-kDa” protein was made *independently* by three different laboratories, each of which, in turn, was coming from a different biological angle. The three and sometimes four independent names given to this molecule –p32/SF-2 (2, 3), gC1qR or p33 (4) HABP1 (5) and C1QBP (6)– are therefore a reflection of the biological function with which this protein was associated at the time of the discovery. Interestingly, although no biochemical or structural information is available to use for comparison with gC1qR, Storrs and his colleagues (7) may, in fact, have been–*as far back as 1981*–the first to independently document the *presence* of a “C1q binding” entity in isolated heart-derived mitochondrial membranes (7). This finding in turn, led them to postulate that the interaction between the mitochondrial “entity” with C1q was responsible for antibody independent activation of the classical pathway that contributed to acute inflammatory response (7). Although, as stated above, there is no amino acid sequence to date to verify if this “mitochondrial entity” is related to, or the same as gC1qR, the known association of gC1qR with the mitochondria, and its ability to activate the classical pathway of complement, give credence to the postulate that the mitochondrial protein described by this group 30 years earlier, and gC1qR may in fact be similar, if not identical.

The major research project of our laboratory has been the identification of cell surface molecules, which interact with C1q. Therefore in 1984, we isolated and identified a 60kDa C1q binding cell surface protein isolated from the *membranes* of Raji cells (8). Consequently, in 1992, while on an NIH-supported sabbatical leave at the University of Oxford with a defined goal to purify and clone the gene for the 60kDa C1q receptor, we also identified a second C1q binding protein from Raji cell membranes, which unlike the 60 kDa protein, was highly negatively charged and had a molecular weight of ~33 kDa on SDS gels (4). When the two proteins i.e., the 60kDa and 33kDa, were compared for their ability to bind C1q, the 60 kDa cell surface protein predominantly recognized the collagen tail of C1q (cC1q)–hence the designation *cC1qR* (9)–whereas the 33 kDa membrane protein recognized mainly the globular heads of C1q (gC1q) and thus named *gC1qR* (9). However, while the N-terminal sequence of cC1qR showed identity with a molecule called calreticulin (CR), whose amino acid sequence was already in the database (10)–hence often referred to as cC1qR/CR (9)–the N-terminal sequence of gC1qR showed *no homology* with *any* protein in the database *at that time*. Therefore, we proceeded to clone the full-length cDNA of gC1qR and found that it consisted of a pre-pro-protein of 282 aa residues (4). Moreover, when the cDNA sequence of the entire gC1qR protein was again searched in the database, it showed identity with a 32 kDa mitochondrial protein called ‘human splicing factor SF-2’ that was identified in 1991 by Krainer and colleagues indicating that it lacked the N-terminal 73aa residues (2). Then, in 1993 Honoré et al. (3), published the cDNA encoding the full-length SF-2 protein which extended beyond the 5’ end of the SF-2 reported earlier (2). This sequence was found to be identical to that of gC1qR (4) indicating that the proteins are the same with the exception of the 73aa N-terminal sequence that was lacking in p32 (2). In 1996, again coming from a different angle, Datta and her group (5), generated a partial sequence of a protein designated hyaluronic acid binding protein (HABP-1), and this too showed identity with SF-2/p32 and gC1qR. Because the initial sequence comparison between gC1qR and SF2 showed the absence of the N-terminal residues 1-73 in SF2, we hypothesize that the SF2/p32 molecule is derived from gC1qR by enzymatic removal of the N-terminal segment containing the start codon. This hypothesis in turn is supported by the finding that fusion of residues 1–81 or 1– 33 of the pre-pro-protein to the N-terminus of the SF-2 protein, directed the fusion protein to the mitochondria (11).

The background information detailed above therefore should help clarify the confusion that is often associated with the various names used to describe this molecule. As is commonly the case in scientific research, the discovery of the same protein was achieved by three different laboratories working independently on different biological systems. Therefore, the use of the names gC1qR/p32/HABP1 to describe this fascinating multifunctional and multicompartmental cellular protein is wholly justified albeit a bit confusing to the reader. The designation of “C1QBP” (C1q-binding protein) (6) however, is strictly reserved to describe the gene that encodes the gC1qR protein and by association, to its homologs SF-2 and HABP-1. In this report, we will use gC1qR for simplicity, and the other designations p32 and HABP-1 will be used within the context in which they are described.

## gC1qR: gene structure and chromosomal location

**Figure 1:**
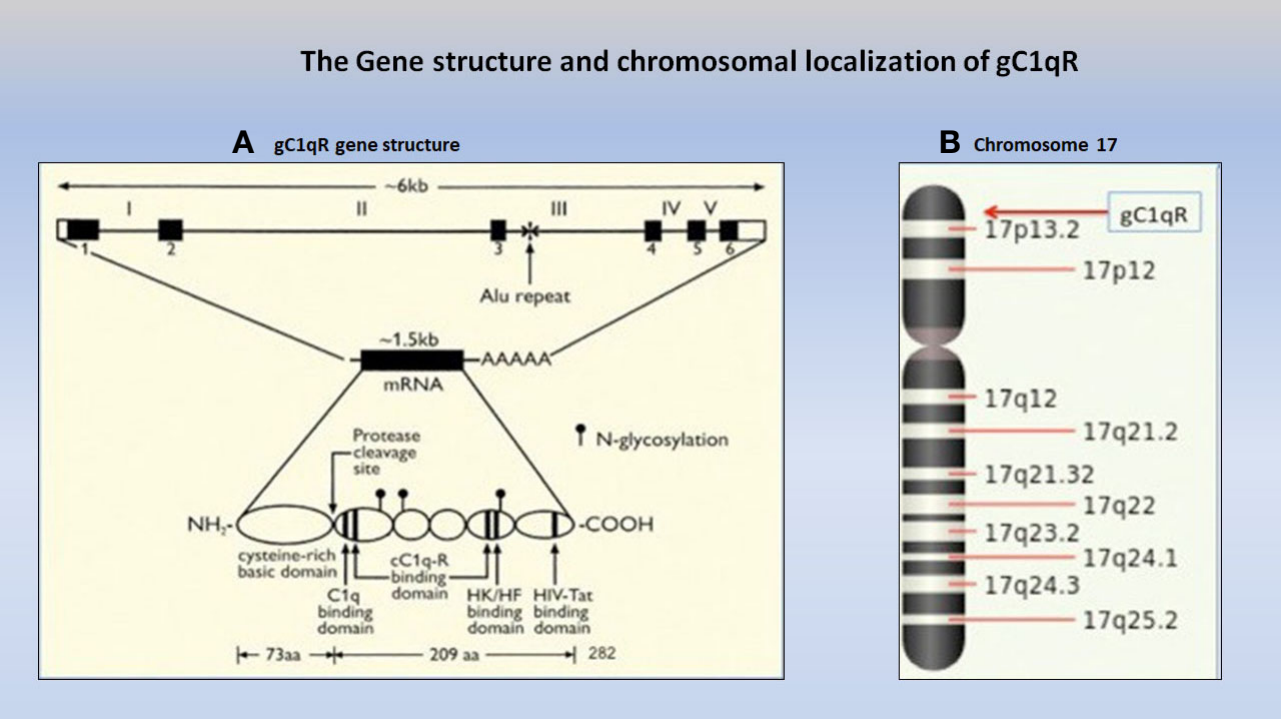
The gC1qR gene **(A)**, which consists of 6 exons and 5 introns (Ref. 6) is localized on the short arm of chromosome 17p13.3 **(B)** together with some of the best-known cancer genes including the two known tumor suppressor genes, p53 and HIC-1.

**Figure 2:**
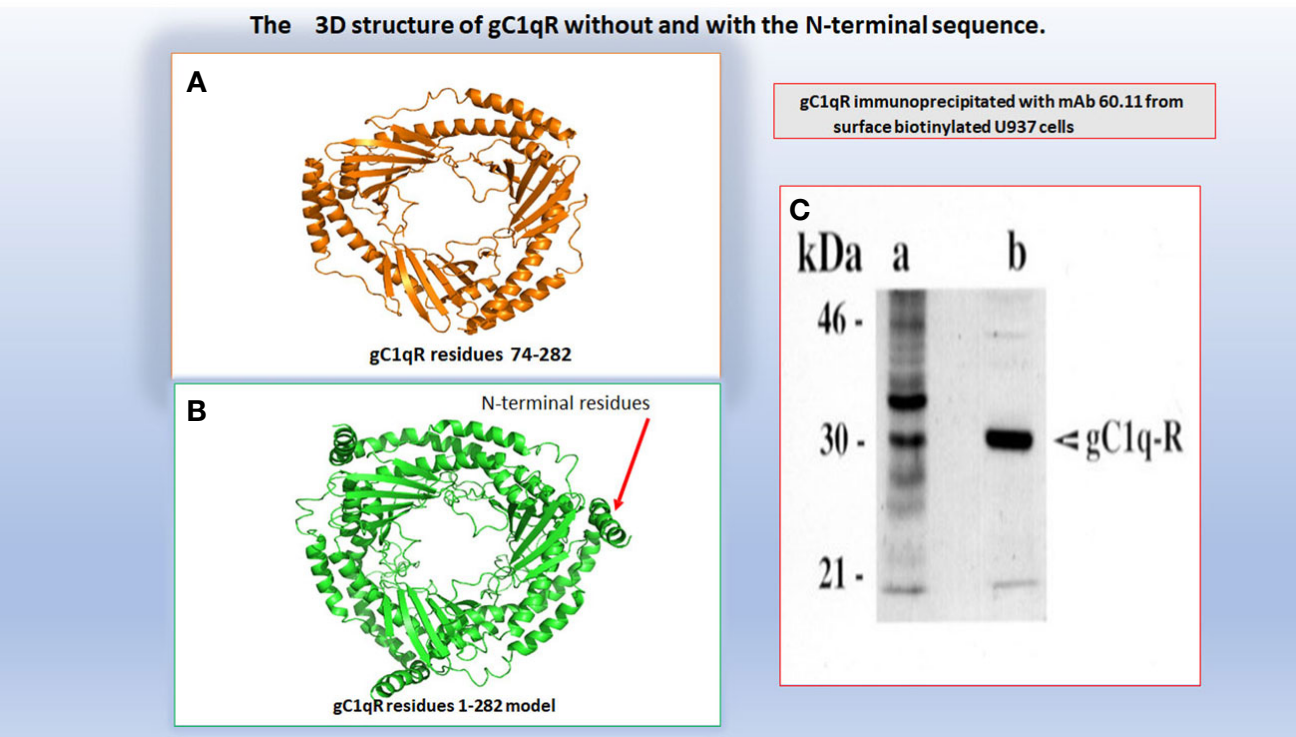
A computer assisted model of the 3D structure of gC1qR without **(A)** and with **(B)** the N-terminal 73aa residues. The original 3D structure of residues 73-282 in **(A)** was solved by Jiang and colleagues and published in PNAS (Ref.12). The molecular weight of gC1qR immunoprecipitated from U937 using mAb 60.11 is shown in **(C)**.

As stated above, the gC1qR/p32/HABP1 molecule, is encoded by the C1QBP gene, which in turn is made up of 6 exons and 5 introns (**Figure 1**) located on the short arm of chromosome 17 at position 17p13.3 (6). Although the translated gC1qR protein migrates at 33 kDa on SDS-PAGE (**Figure 2**), it has a molecular weight of ~96.2 kDa by gel filtration indicating that the mature molecule is a homotrimer of three identical chains (4). A computer model comparing the crystal structure of p32 (12), with that of the full-length 33 kDa protein is depicted in **Figure 2**, in order to show the localization of the 73aa-long N-terminal segment found in the whole molecule but absent in the SF-2 protein that was originally used for crystallography (12).

The gC1qR gene is highly conserved throughout species. Comparison of the amino acid sequence of the human to that of rodent gC1qR shows 89.9% identity, whereas comparison of the rat and mouse DNA shows 97.56% identity indicating that this molecule is evolutionarily highly conserved throughout species i.e., from arthropods to mammals (13).

## gC1qR: localization and cellular functions

**Figure 3:**
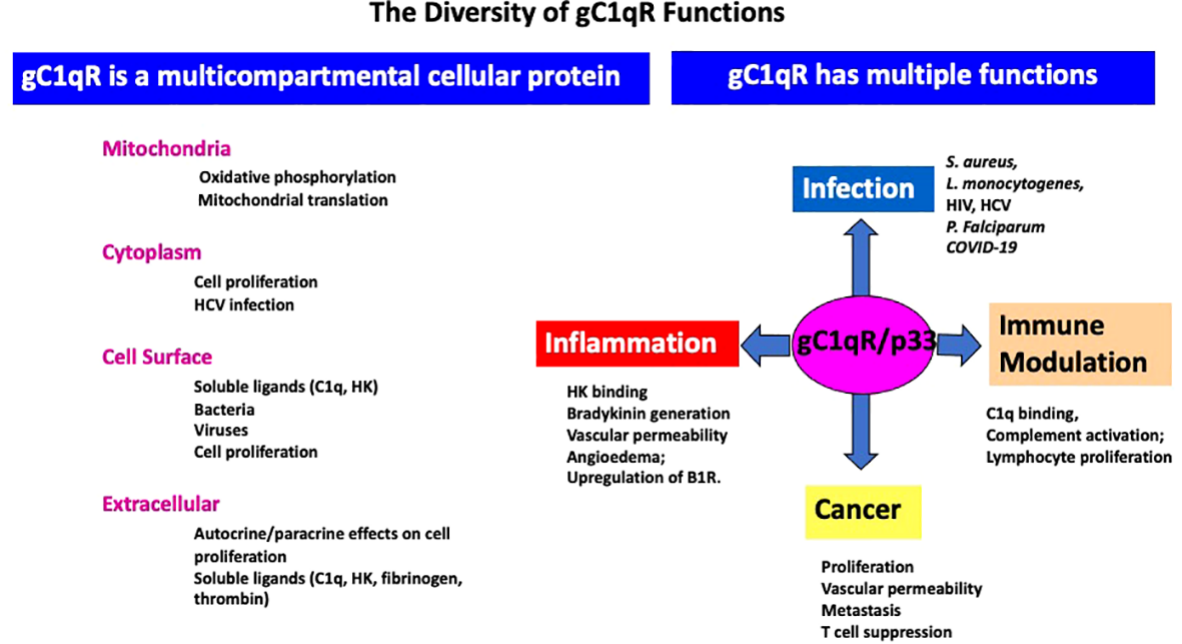
The diversity and growing list of the role of gC1qR in–infection, inflammation, immune modulation, and cancer–and the different cellular compartments it is located –such as the mitochondria, cytoplasm, cell surface and extracellular milieu- is summarized in this figure. In addition to activation of the complement system, gC1qR is also a major receptor for high molecular weight kininogen (HK) and triggers the activation of the kinin kallikrein system (KKS) leading to the generation of bradykinin, one of the most potent vasoactive molecules known and which is the cause of angioedema. In addition, gC1qR in the pericellular milieu can also upregulate the expression the inducible bradykinin receptor 1 (B1R).

**Figure 4:**
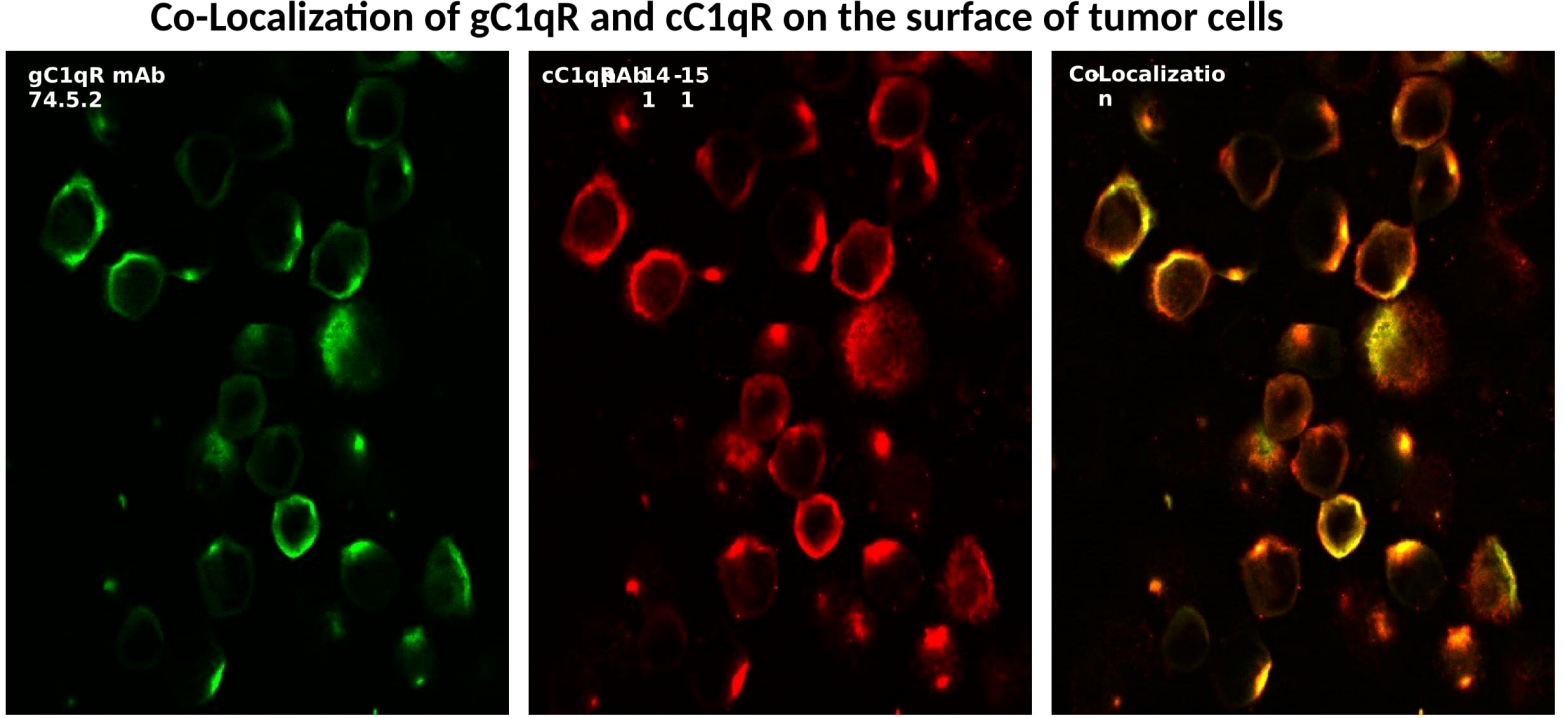
Surface expression and co-localization of gC1q-R and cC1qR on SkBr cells. The cells were first were grown on cover slips with PBS containing 0.1%BSA and 1mg/ml Fc fragments to block the FcRs (1 h,37° followed by incubation with either control antibody (MOPC-21), or mAb 74.5.2 or pAb to cC1qR as described earlier (Ref. 28).

**Figure 5:**
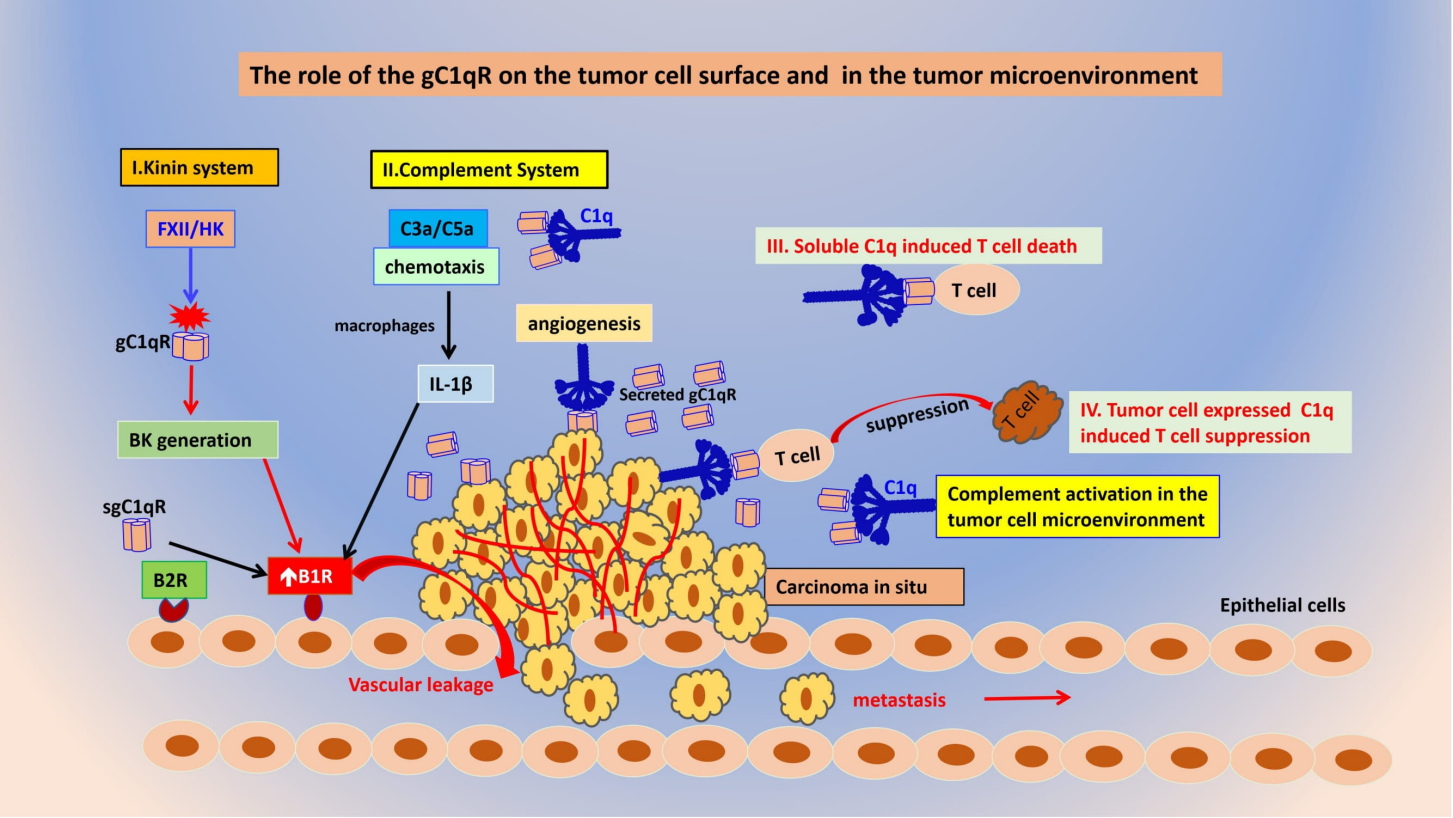
The role of gC1qR and C1q on the tumor cell surface and in the tumor microenvironment. Tumor cell released gC1qR can activate both the kinin (I) and complement systems (II) thereby releasing vasoactive peptides such as BK and C3a and C5a that play a role in B1R generation resulting in vascular leakage that favors metastasis. Both soluble C1q (III) and tumor cell expressed (IV) can cause suppression of cytotoxic T cells in a manner similar to PD-L1 and PD-1.

As a ubiquitously expressed multifunctional and multicompartmental protein found in the mitochondria, the cytosol, the cell membrane, as well as in inflammatory fluids (2–5), gC1qR is expected–and indeed has been shown–to participate in a plethora of distinct localized functions as summarized in **Figure 3**. Furthermore, because of its role in cell metabolism, proliferation, as well as cellular homeostasis, it is considered to be an essential protein for host survival. It is not therefore surprising to find that p32-knockout mice exhibit “mid-gestation lethality” (14). In addition to its mitochondrial localization, there is also plenty of experimental evidence which shows gC1qR localization at specific extramitochondrial locations in normal tissues (15). Some of the emerging functions of gC1qR include involvement in C1q-mediated clearance of apoptotic self-molecules as well as in immune regulation of T and B lymphocytes and maturation of dendritic cells (16–18). Moreover, gC1qR that is elaborated during cell stress participates in the inflammatory response for host protection against pathogens and as such is an important component of the innate immune response mechanism. The full-length gC1qR is present on the cell surface co-localized with cC1qR as shown by staining of cells with a monoclonal antibody to gC1qR and a polyclonal antibody raised against a synthetic peptide corresponding to cC1qR residues 141-151 (**Figure 4**). Malignant cells, which overexpress gC1qR are also able, to release–probably by a proteolytic cleavage that involves the metalloproteinases MT1-MMP (19) or other heretofore unidentified enzyme(s)–a soluble form of gC1qR into the pericellular milieu, which in itself is a useful diagnostic molecular marker of cell proliferation and malignancy. More importantly however, the secreted or released form of gC1qR has been shown to play a diversity of functions including activation of potent pro-inflammatory pathways such as the complement and kinin systems (**Figure 5**), both of which generate activation peptides that play important roles in, cell proliferation, metastasis, and cancer cell survival (20–22).

## The role of gC1qR in cancer

In addition to the C1qBP gene, chromosome 17 (**Figure 1**), is also home to some of the most powerful oncogenes, including HER2, TOP2A, TAU, as well as to tumor suppressor genes including BRCA-1, p53, and HIC-1, that are known to be associated with several types of cancer, including breast cancer (BRCA-1).

Therefore, by virtue of its localization with these oncogenes, we hypothesized that the gC1qR gene may also be involved in carcinogenesis. Not surprisingly, we found that gC1qR is not only overexpressed in most cancer cell types but is actively involved in tumorigenesis at various levels suggesting that gC1qR expressing tumor cells are more aggressive and have a better survival rate than non-expressing cells (23, 24). In grafted mice for example, A549 cells depleted of gC1qR had reduced tumorigenic and metastatic activity (23). Additionally, studies of lung carcinoma A549 cells showed cell surface gC1qR to be involved in tumor progression through its role as a key regulator of lamellipodia formation via receptor tyrosine kinase activation (23). Moreover, overexpression of gC1qR has also been associated with decreased overall patient survival and decreased progression free survival in serous ovarian adenocarcinoma and endometrial cell cancer (23). In breast cancer, cell surface expressed gC1qR has been identified as a receptor for the tumor homing peptide LyP-1, targeting tumors for lymphatic spread, and metastasis (25). Therefore, overexpression of gC1qR may serve not only as a prognostic indicator but also as a potential novel target for therapeutic intervention in a wide range of cancer types. The biological significance of gC1qR is further demonstrated by the fact that gC1qR KO in mice is embryonic lethal (14, 26). These mice exhibited mid-gestation lethality associated with severe developmental defect of the embryo (26). Primary embryonic fibroblasts isolated from gC1qR KO embryos, showed severe dysfunction of the mitochondrial respiratory chain, because of severely impaired mitochondrial protein synthesis. Furthermore, a biallelic mutation in the C1qBP gene causes severe neo-natal or later-onset cardiomyopathy that is associated with respiratory deficiency (14, 26).

Most if not all malignant cells are known to overexpress both C1q and gC1qR on their surface in a manner that assists and enhances tumor cell growth and metastasis through mechanisms that are still being studied. In general, the gC1qR protein is found inside the cell, and on the cell membrane. However, most proliferating cells also secrete gC1qR into the pericellular milieu. The secreted gC1qR, in turn can participate in a wide range of pro-proliferative activities that collectively enhance tumor cell survival and growth (19). A tumor cell is a “modified self” and as such expresses “modified-self antigens” that would help it survive in an otherwise hostile immunologic milieu. These “modified-self antigens” in turn are seen by the immune system as “foreign” antigens and induce the production of circulating autoantibodies. For example, the triple negative breast cancer cell line, MDA-MB-231, expresses PI3K and P53 antigens, both of which are known to induce the production autoantibodies (27). Theoretically therefore, these “autoantibodies”, should bind to the corresponding antigen on the tumor cell and trigger complement activation leading to tumor cell lysis. However, *this is not the case* (28). One of the major reasons being that the tumor cell is protected by a “*molecular shield*” in the form of secreted gC1qR, which binds to the globular heads of the oncoming plasma C1q, thus preventing it from binding to the immune complexes on the cell surface (**Figure 5**). More importantly, the secreted gC1qR can also serve as a molecular anchor for the assembly and activation of both the complement and the kinin-kallikrein systems in a manner that benefits the overall health of the cancer mass. For example, soluble gC1qR can recruit C1q, as well as FXII, high molecular weight kininogen (HK), and prekallikrein (PK) to the pericellular milieu (22). Activation of both pathways (**Figure 5**) in turn, generates powerful cleavage products such as C3a and C5a from the complement system, and bradykinin (BK) from the kinin system, that together play a major role in vasodilation, metastasis, and inflammation (22). Moreover, the secreted form of gC1qR is also able to upregulate the expression of the inducible bradykinin receptor 1 (B1R) in an autocrine manner (20), leading to enhanced vascular leakage and thus opening intracellular “gates” that allow tumor cell escape and metastasis to distal sites. Because proliferating tumor cells are dependent on angiogenesis for survival, progression, as well as escape to secondary sites for metastatic invasion, mAbs that inhibit these processes have now become *prime targets* for the development of anti-cancer therapy. This hypothesis is supported by our earlier findings that the proliferation of several cultured tumor cell lines could be inhibited in the presence of either mAb 60.11 that recognizes the C1q binding site on the tumor cell anchored gC1qR, or by a polyclonal antibody that recognizes the gC1qR binding site on the A-chain of C1q (28) indicating that both surface expressed gC1qR and C1q play an important role in cell signaling that favors proliferation.

## The C1q-gC1qR axis as a novel checkpoint inhibitor

Unlike most of the complement proteins, which by and large, are synthesized in the liver, the C1q molecule is mostly synthesized extrahepatically by different cell types, including, monocytes, macrophages, dendritic cells, fibroblasts, and endothelial cells (29–33). C1q is a member of the tumor necrosis factor (TNF) superfamily of proteins and plays an important role in inflammation as well as in apoptotic cell removal (33). In general, the TNF family of proteins bind extracellularly to cysteine-rich receptors, to induce a clustering of the receptors that trigger the intracellular apoptotic cascade (34). The TNF proteins–including C1q–are therefore important mediators of inflammation, immune responses, and cytotoxicity through interaction with the TNF-R55 and the TNF-R75 cell-surface receptors (34–37), in a manner similar to the C1q-gC1qR interaction.

In general, simultaneous overexpression of gC1qR and C1q is a hallmark of epithelial derived malignancies. The significance of gC1qR in tumorigenesis is further demonstrated by the fact that high gC1qR expression by breast cancer cells is associated with poor survival due to enhanced proliferation, inflammation, and vascular permeability (1). We have shown previously, that membrane-associated gC1qR and C1q are both pro-proliferative, since incubation of C1q with T cells, which express gC1qR, induces an anti-proliferative response (38). Furthermore, co-culture of the breast cancer cell line, SkBr3 with C1q or its globular heads resulted in inhibition of cell growth with the ghA heads being more efficient than the ghB or ghC domains (28). Moreover, co-culture of the SkBr3 cells with anti-C1q or an antibody directed against the ghA site of gC1qR also resulted in inhibition of cell proliferation (28). Although T cells express both cC1qR and gC1qR on their surface, the C1q, which is anchored on the tumor cell via its collagen tail, can only bind to gC1qR expressed on the oncoming cytotoxic T cells–via its globular heads. However, C1q in plasma or released from cells into the tumor site, can potentially bind to either cC1qR via its collagen region or to gC1qR via its globular heads, although the affinity of C1q for gC1qR may favor this interaction. It is not therefore surprising to find that the proliferative function of tumor cell surface expressed gC1qR can be abrogated by blockade of its C1q binding site, while the proliferative function of cell surface C1q, is abrogated by blockade of the gh (globular head) domains, again confirming that the pro-proliferative function of C1q resides in its *gh domains*, with the ghA domain playing a central role (28).

As a ubiquitously expressed multi-compartmental cellular protein involved in a diversity of functions, gC1qR is recognized as an essential protein for cell survival. In support of this hypothesis is the finding that the biallelic mutation in the C1QBP gene results in deficiency in oxidative phosphorylation (39, 40). Furthermore, homozygous mutation in C1QBP results in progressive external ophthalmoplegia (PEO) and mitochondrial myopathy (41). Recent experiments have also shown that mitochondrial gC1qR supports glycolysis and enhances cell survival in an anaerobic tumor environment (40–42). Almost every cancer cell type examined to date has been shown to express C1q anchored on its surface with the globular heads displayed outwardly for maximal recognition and binding (27). Our hypothesis is that the surface expressed C1q is able to inhibit and silence the oncoming tumor infiltrating cytotoxic T cells by binding to the gC1qR expressed on their surface in a manner similar to the PD-L1–PD1 checkpoint inhibition with the tumor cell expressed C1q serving the role of PD-L1 and the gC1qR on the cytotoxic T cells serving the role of PD-1. On the basis of these observations, we propose that the C1q-gC1qR axis is a *novel checkpoint inhibitor* that is a potential target for antibody-based or small molecular weight-based therapy. To prove this hypothesis, we designed and performed proof-of-concept preliminary studies, which were published earlier (1) and some of the findings are summarized below.

## Preliminary proof-of-concept studies in animal models

Data accumulated to date show that gC1qR fulfills very important homeostatic functions and as such plays a role in both innate and adaptive immunity (42). Previous experiments have shown that targeting gC1qR-expressing cells with C1q induces an antiproliferative response (38). Because high gC1qR expression by most cancer cells is associated with poor survival, gC1qR has become a potential prime target for the development of anti-cancer therapy (43, 44). Indeed, we are very much aware that targeting such a ubiquitously expressed, multifunctional, and multicompartmental molecule, would theoretically present an undesirable collateral risk since it has the potential to lead to deleterious off-target blockade of important biological functions. For example, gC1qR blockade with monoclonal antibodies (mAbs)– i.e. mAb 60.11, which recognizes the C1q site on gC1qR and mAb 74.5.2, which recognizes the HK site on gC1qR–may diminish the host response to local infection such as decreased migration of neutrophils and macrophages into sites of infections as a result of decreased local production of C3a (chemoattractant) (mAb 60.11) and C5a (mAb 60.11), as well as bradykinin (mAb 74.5.2) (vascular permeability). Since mAb 74.5.2 leads to inhibition of bradykinin generation, its side effects are expected to be similar to those encountered with the current FDA approved therapies for angioedema, including C1 INH therapy, kallikrein antagonists and bradykinin receptor blockers (45–47).

To date, the only information addressing the dose required to adequately block the gC1qR molecule *in vivo* comes from our previous studies performed in a rat model of infective endocarditis using *S. aureus* (29). In this model, both mAb 74.5.2 and 60.11 were administered intraperitoneally (IP) at 100 mg/Kg. Circulating levels of 100 µg/mL were detected by ELISA and the animals tolerated this dose until they were sacrificed 3 days later. The results showed that while a significant reduction in *S. aureus* colonization of vital organs was observed when compared to untreated animal cohorts, there was no observable cytotoxic off-target side effects as a consequence of antibody treatment (29). Encouraged by these results, we tested the effect of mAb treatment in a mesothelioma model, which is an aggressive cancer of the serous membranes with poor prognosis even after combination therapy consisting of surgery, radiotherapy, and platinum-based chemotherapy (44). Targeted therapies, including immunotherapies, have reported limited success, suggesting the need for additional therapeutic targets. Therefore, we designed *in vivo* studies, in a murine orthotopic xenotransplant model using the biphasic mesothelioma cell line MSTO-211H (MSTO). The results of these animal studies demonstrated an even greater reduction in MSTO tumor growth (50% inhibition) in mice treated with mAb 60.11 compared to control mAbs targeting different regions of the gC1qR molecule (44). Moreover, immunohistochemical studies of resected tumors revealed increased cellular apoptosis by caspase 3 and TUNEL (Terminal deoxynucleotidyl transferase dUTP nick end labeling) staining, in 60.11 treated tumors compared to controls, as well as impaired angiogenesis by decreased CD31 staining (24, 44).

**Figure 6:**
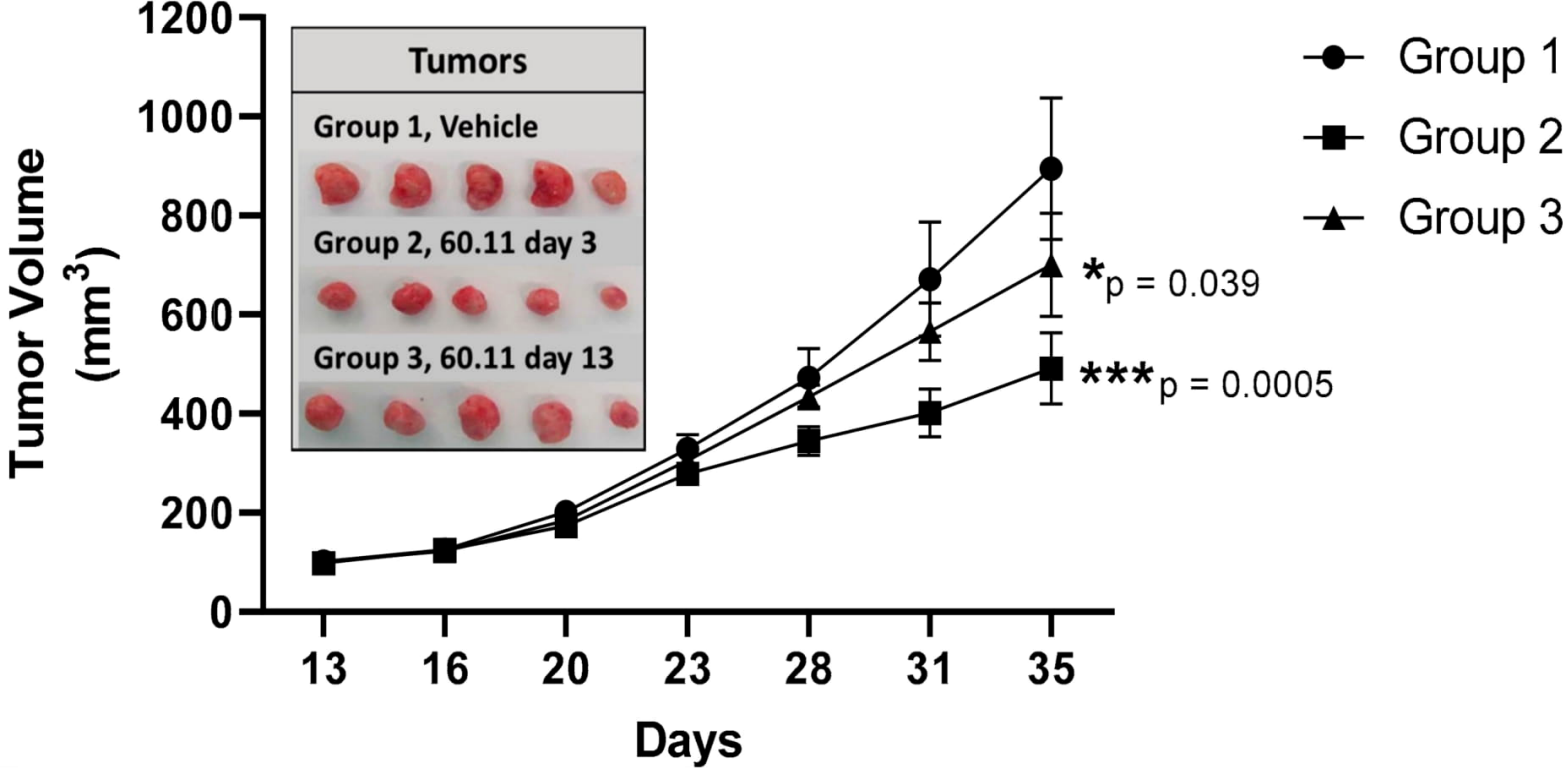
Tumor development in vehicle control and 60.11-treated mice. gC1qR therapy with 60.11 antibody inhibits MDA231 cell proliferation. Tumor volumes of vehicle-treated control mice (group 1) and mice treated with 60.11 antibody are presented over time (35 days). 60.11 therapy was initiated either three days after MDA231 cell implantation (group 2) or on day 13, when tumor volume had reached approximately 100 mm3 (group 3). Mean and standard deviation (SD) of tumor volume is shown for each treatment group (n = 5 animals per group). P values were determined by Student t-test. (*) designates statistically significant differences in tumor volume between control and treatment groups (p < 0.05). Images of individual tumors resected at study termination are shown in the inset [adapted from *Antibodies* (2020) 6: 9(4) 51)]. *=p<0.05; ***=p<0.001.

Therefore, encouraged by the results obtained on the rat and mouse models, we performed proof-of-concept studies in a mouse model for triple negative breast cancer to demonstrate the therapeutic potential of targeting gC1qR with an antibody directed against the C1q binding site of gC1qR (**Figure 6**). As discussed extensively in our previously published paper (1), this approach produced very positive and encouraging data. Briefly, MDA-MB-231 breast cancer cells were first injected into the mammary fat pad of athymic nu/nu mice. The mice were separated into three study groups: mAb 60.11 treated, vehicle treated, or mAb 60.11 treated after the tumor mass had already reached 100 mm^3^ (1). At the end of the experiment, which is essentially after the tumor size had reached 100 mm^3^, tumor pieces were excised from each group and analyzed using immunochemical staining.” Increased apoptosis was found in the mAb treated group as evidenced by caspase 3 and TUNEL staining (1). More importantly, there was no evidence of pathologic or overt toxicity on off target tissues or cell damage and nor were appreciable histologic changes in vital organs as a consequence of antibody treatment (1). This is indeed the first proof-of-concept study demonstrating that targeting gC1qR with antibody directed against the C1q binding site inhibits MDA-MB-231 breast cancer cell proliferation *in vivo* without *significant toxicity* over a 30-day treatment course. This suggests that gC1qR represents a potential novel and effective therapeutic target against triple negative breast cancer.

Although these are indeed very limited studies using one type of cancer, the three animal studies performed to date, using mAbs targeting different regions of gC1qR, should mitigate the legitimate concern that such a ubiquitously expressed molecule could be targeted for therapy without consequential side effects (1, 44). In general, the net balance of gC1qR and C1q expression in the tumor microenvironment is critical for tumor cell survival and progression. For example, while overexpression of gC1qR suppresses the tumor inhibiting role of C1q and promotes tumor proliferation in multiple myeloma (48), gC1qR overexpression in some tumor cells such as mesothelioma is associated with better prognosis and survival when compared to breast cancer cells particularly when the tumor microenvironment is infiltrated with gC1qR expressing CD8 lymphocytes (24). The reason for this is because, while mesothelioma cells express cytosolic, cell surface, and soluble gC1qR, they *do not* express cell surface C1q (24). In the absence of C1q therefore, there would be no C1q-gC1qR induced *checkpoint inhibition* thus resulting in enhanced tumor cell killing by infiltrating CD8 cells.

Taken together, the experiments summarized herein identify gC1qR as a potential novel therapeutic option not only against breast cancer but potentially against most cancer types that overexpress gC1qR as part of tumorigenesis. Moreover, since the expression of C1q on tumor cells also plays a critical role in tumor cell survival through interaction with and suppression of gC1qR expressing T cells, an antibody that targets the gC1qR site on gC1q could also be another option to disrupt the C1q-mediated T cell suppression Therefore, we believe that the development of anti-gC1qR therapy alone or in combination with anti-C1q could enhance presently available therapies to treat not only breast cancer, but other types of cancer as well.

## Concluding remarks

Since the discovery of gC1qR in the early 1990s, we have accumulated a large body of structural and functional information, which justify its unique multi-functional and multi-compartmental properties. In this report, we have attempted to highlight the critical areas in cancer, which are not only ripe for robust and focused investigation over the near and intermediate future but, by virtue of their pathologic significance, have drawn attention as potential targets for antibody-based or small molecule-based therapy in inflammation, infection, and cancer. With unexpected discoveries coming out from different laboratories throughout the world, the future of gC1qR studies is very bright indeed. More importantly however, it is hoped that the knowledge gained from these studies will serve as a template for the design of therapeutic modalities not only for cancer but also for a long list of infectious and inflammatory diseases in which gC1qR has been shown to be heavily involved. Per force, *in vivo* studies are required to address these questions and understand the pharmacokinetics and dynamics of gC1qR antibody therapy, as well as its toxicologic effects. We plan to conduct these studies with humanized antibodies in murine models in our institution’s Antitumor Assessment Core. The availability of humanized antibodies will allow us to perform toxicity studies in mice with emphasis on the antiproliferative effects of mAb 60.11, visualizing human antibody on host cells and tissues, performing immunohistochemical staining for apoptotic cell death, as well as following blood cell counts to evaluate effects on hematopoiesis. The development of systemic inflammation may also be visible histologically. If animal studies indicate significant toxicologic effects of systemic 60.11 antibody administration, we could consider local therapy administered to defined anatomic spaces. For example, instillation into the bladder as a treatment for bladder cancer, or into the pleural space for potential treatment of mesothelioma could be studied.

## Author contributions

BG: Conceptualization, Funding acquisition, Project administration, Supervision, Validation, Writing – original draft. MZ: Writing – review & editing. AF: Writing – review & editing. MD: Data curation, Writing – review & editing. TR: Data curation, Writing – review & editing. PJ: Writing – review & editing. AS: Writing – review & editing. JW: Conceptualization, Investigation, Methodology, Writing – review & editing. MS: Data curation, Writing – review & editing. EP: Conceptualization, Data curation, Investigation, Methodology, Writing – review & editing.
